# ecRESCUE: a novel ecDHFR-regulated RESCUE system with reduced RNA off-targeting activity

**DOI:** 10.1186/s12964-021-00759-2

**Published:** 2021-07-31

**Authors:** Yihan Wang, Guo Li, Xiangyang Li, Yuzhe Wang, Xingxu Huang, Xiaoxiang Hu, Jianen Gao

**Affiliations:** 1 National Research Institute for Family Planning, Beijing, 100081 China; 2 Graduate School of Peking, Union Medical College, Beijing, 100730 China; 3 State Key Laboratory of Agrobiotechnology, China Agricultural University, Beijing, 100193 China; 4 College of Biological Sciences, China Agricultural University, Beijing, 100193 China; 5 School of Life Science and Technology, Shanghai Tech University, Shanghai, 200031 China; 6 College of Animal Science and Technology, China Agricultural University, Beijing, 100193 China; 7 CAS Center for Excellence in Molecular Cell Science, Shanghai Institute of Biochemistry and Cell Biology, Chinese Academy of Sciences, University of Chinese Academy of Sciences, Shanghai, 200031 China

**Keywords:** Dihydrofolate reductase destabilization domain, ecRESCUE, Off-targeting activity, RESCUE system, RNA base editing

## Abstract

The currently available RESCUE RNA base editing system demonstrates considerable potential for the treatment of genetic diseases at the transcriptional level. However, the relatively high incidence of off-target events hampers the precise RNA editing, thereby limiting its use in the clinical setting. This study describes a new RNA base editing method, named ecRESCUE, which utilizes inducible stabilization of the protein ecDHFR DD fused at the C-terminal of the original RESCUE system. In vitro experiments in 293T cells showed that the ecRESCUE editor markedly reduced the incidence of off-target single nucleotide polymorphisms without affecting the RNA A-to-I and C-to-U base editing efficiency. Altogether, these results demonstrate that the inducible ecRESCUE system represents an attractive approach to regulate and improve the outcome of the available RNA base editor with reduced off-targeting activity.

**Video Abstract**

**Supplementary Information:**

The online version contains supplementary material available at 10.1186/s12964-021-00759-2.

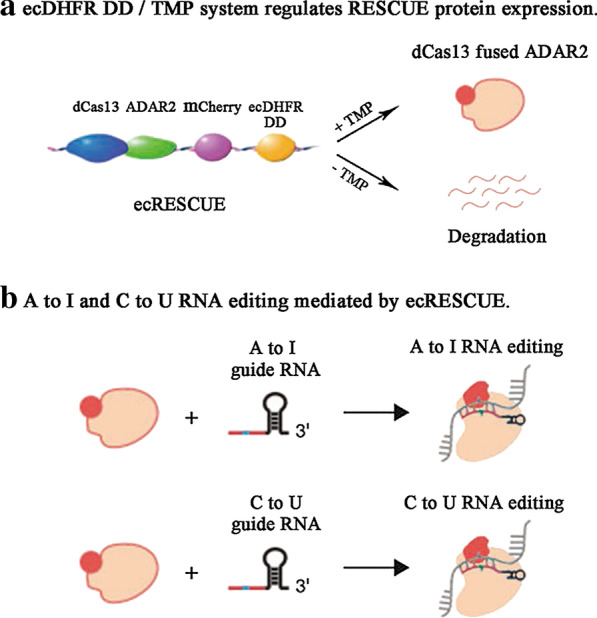

## Introduction

Researchers have recently developed RNA editors that use deactivated Cas13 from *Riemerella anatipestifer* (dRanCas13b) and adenosine deaminase acting on RNA (ADAR) enzymes to catalyze the hydrolysis of adenosine (A) to inosine (I) in mammalian cells using RNA as template. The first RNA base editor, named RNA Editing for Programmable A to I Replacement (REPAIR), does not require strict sequence constraints and can be used to mediate all the A-to-I base editing of the full-length transcripts [1]. By directly evolving ADAR2 into a cytidine deaminase, the RESCUE (RNA editing for specific cytidine to uridine exchange) RNA editor has been developed, which could be used to mediate multiple both A-to-I and C-to-U base editing [2]. Hence, RNA editors represent a useful tool for conducting genetic disease treatment and gene function study at the transcriptional level. Nonetheless, A-to-I and/or C-to-U off-target single nucleotide polymorphisms (SNPs) induced by the REPAIR and RESCUE systems hamper precise RNA editing.

The ecDHFR DD (*Escherichia coli* dihydrofolate reductase destabilization domain) has been used to regulate interest protein expression in mammalian cells [3–5]. The destabilizing domain involved in ecDHFR is a structurally unstable as well as unfolded domain. When this area is combined with proteins, it can be made more unstable, or vice versa, by intervention such as adding missense mutations. DD is derived by ecDHFR and stabilized by the DHFR inhibitor TMP. The high stabilization ability of TMP gives the system a dynamic range. The absence of TMP allows stabilization reversal. The most important point is that the ligand TMP works on its alone and does not require dimerization with a second protein. TMP has properties such as commercial availability, cheapness, and good pharmacodynamics. These features facilitate the establishment of an experimental system in vivo. Moreover, TMP has little off-target effect in mammalian cells and triple inhibition of ecDHFR. TMP's crossing the blood–brain and placental barrier is advantageous for modulating proteins in the central nervous system and early stages of development. In this study, a novel TMP-induced ecDHFR DD system for regulating the effective expression of dRanCas13b and ADAR2 proteins was developed, to further reduce the off-target events of the RESCUE RNA base editor. Ideally, the new RESCUE RNA base editor has low A > I and C > U off-targets, which can be used for precisely pathogenic mutation treating and RNA biology researches.

## Materials and methods

### Plasmid construction

The construct pC0078 RESCUE (#130661) [2] was obtained from Addgene (https://www.addgene.org/). To construct ecDHFR-mCherry and mCherry-ecDHFR, we initially amplified the ecDHFR DD and mCherry expression plasmids, including the colinear homologous segments. Subsequently, the ecDHFR DD was fused to the N- or C-terminal of *mCherry* using the ClonExpressII One Step Cloning Kit (C112-01, Vazyme Biotech, Nanjing, China). Similarly, the mCherry expression RESCUE system (mCherry-RESCUE) was obtained by performing fusion of *mCherry* with the C-terminal of ADAR2 obtained from the pC0078 RESCUE backbone. Thereafter, the ecRESCUE plasmid was constructed by performing fusion of ecDHFR DD with the C-terminal of *mCherry* obtained from the mCherry-RESCUE expression backbone. The sgRNA expression plasmids, which expressed the GFP fluorescence protein, were cloned into two *Bbs*I restriction enzyme digestion sites (Additional file 1: Table S1). The complete base sequence information of all plasmids can be found in supplementary material (Additional file 1: Table S4 and Table S5).

### Cell culture

293T cells were cultured in the Dulbecco’s Modified Eagle Medium (DMEM; Gibco, Waltham, MA, USA), maintained at 37 °C with 5% CO_2_ under humid conditions and supplemented with 10% fetal bovine serum (FBS; Gibco, Waltham, MA, USA).

### Cell transfection

293T cells were seeded in poly-d-lysine-coated 48-well plates and maintained at approximately 60–70% confluence. The cells were transfected with 500 ng of mCherry-ecDHFR or mCherry expression plasmids, which mCherry was used as a fluorescent tracer, using 1.5 µL EZ Trans Reagent diluted in 25 µL DMEM. The diluted EZ Trans Reagent was added into the diluted DNA solution; the mixture was mixed gently and incubated for 15 min at 20–25 °C to allow the formation of DNA-EZ Trans Reagent complexes. Thereafter, the DNA-EZ Trans Reagent complexes were directly added to each well containing cells and mixed gently by rocking the plate several times.

The culture medium was carefully removed 6 h post-transfection and 0.3 mL of the complete growth medium was added to the wells. At 24 h post-transfection, different doses of TMP (0, 1, 2, 4, 6, or 8 ng/μL) were added to the medium. Fluorescence imaging was conducted among different time points.

### Statistical analysis after fluorescence imaging

All fluorescence images were acquired using the Nikon Ti-E microscope (Tokyo, Japan), and the Image J software (U. S. National Institutes of Health, Bethesda, Maryland, USA) was used to analyze the mCherry integrated density as follows:

$$Average\;fluorescence\;intensity = \frac{ Integrated\; density\;of\;red\;image}{{Area\;of\;bright\;image}}$$

### RNA isolation and RT-PCR

Total RNA of the 293T cells expressing RESCUE and ecRESCUE were extracted by using the TRIzol reagent (Thermo Fisher Scientific, Waltham, MA, USA). cDNA was synthesized using oligo d(T) primers and used as the RT-PCR template for the detection of *dRanCas13b*, *ADAR2*, and mCherry expression. Primers used for conducting RT-PCR are listed in supplemental information (Additional file 1: Tables S2 and S3).

### Endogenous mRNA editing in 293T cells

Before transfection, 293T cells were cultured in 24-well plates and maintained at approximately 60–70% confluence. The DNA transfection mixes included 330 ng sgRNA expression plasmids in each well, along with 660 ng of ecRESCUE or RESCUE expression plasmids. Overall, 0.99 μg DNA and 4.5 µL EZ Trans Reagent diluted in 40 µL DMEM were added to each well. The diluted EZ Trans Reagent was added into the diluted DNA solution; the mixture was mixed gently and incubated for 15 min at 20–25 °C. The mixture was directly added to each well containing cells and mixed gently by rocking the plate several times. The complexes were then removed 6 h post-transfection and 0.5 mL of the complete growth medium was added to the cells. At 48 h post-transfection, 2 × 10^4^ GFP-positive cells were collected by fluorescence activating cell sorting and total RNA was extracted using the TRIzol reagent (Thermo Fisher Scientific). cDNA was acquired by HiScript III 1st Strand cDNA Synthesis Kit (+gDNA wiper) (R312-01, Vazyme). Thereafter, the editing segments of the endogenous mRNA targets were amplified by PCR and separated by performing agarose gel electrophoresis. The gel products with the correct size were purified and sequenced using the amplification primers. The RNA editing efficiency was assessed using the Edit R tool (https://moriaritylab.shinyapps.io/editr_v10/). [6]

### Whole-transcriptome sequencing

The RNA off-targets released by RESCUE system may affect the endogenous RNA expression, because the point mutation locates in different site, such as inducing the STOP codon formation, and the off-targets may affect the miRNA expression and circRNA-miRNA-mRNA network. The over-expression of ADAR2 in the RESCUE system is responsible for the numerous RNA off-targets. In this study, we reduced the effective time of ADAR2 by the use of the ecDHFR/TMP regulation system to reduce the RNA off-targets. Three sites (*KRAS*, *NFKB1*, and *NRAS*) were selected to detect the RNA off-target events across the transcriptome. sgRNA expression plasmids targeting *KRAS*, *NFKB1*, and *NRAS* were co-transfected with each of two RNA editors, RESCUE or ecRESCUE, into 293T cells seeded in 6-well plates. Two biological repeats were set up for each sample. More than 5 × 10^5^ GFP-positive cells for each sample were sorted by fluorescence-activated cell sorting and approximately 5 μg total RNA of each sample was extracted and subjected to RNA sequencing.

RNA sequencing was performed using the Novaseq system (Illumina, San Diego, CA, USA) by Novogene (Beijing, China). NEBNext® UltraTM RNA Library Prep Kit for Illumina® (NEB, USA) was used to generate the sequencing libraries. TruSeq PE Cluster Kit v3-cBot-HS (Illumia) was used to perform the index-coded samples’ clusters. Then the library preparations were sequenced on an Illumina Novaseq platform.

Two main software were used to analyze the off-target SNPs, including GATK2 (v3.7) for performing SNP calling and SnpEff for annotation the variable sites. The RNA off-target SNPs analysis method for the RESCUE system was performed in accordance with methods reported by Abudayyeh et al. [2].

## Results

### mCherry was well-regulated by fused ecDHFR DD

**Fig. 1:**
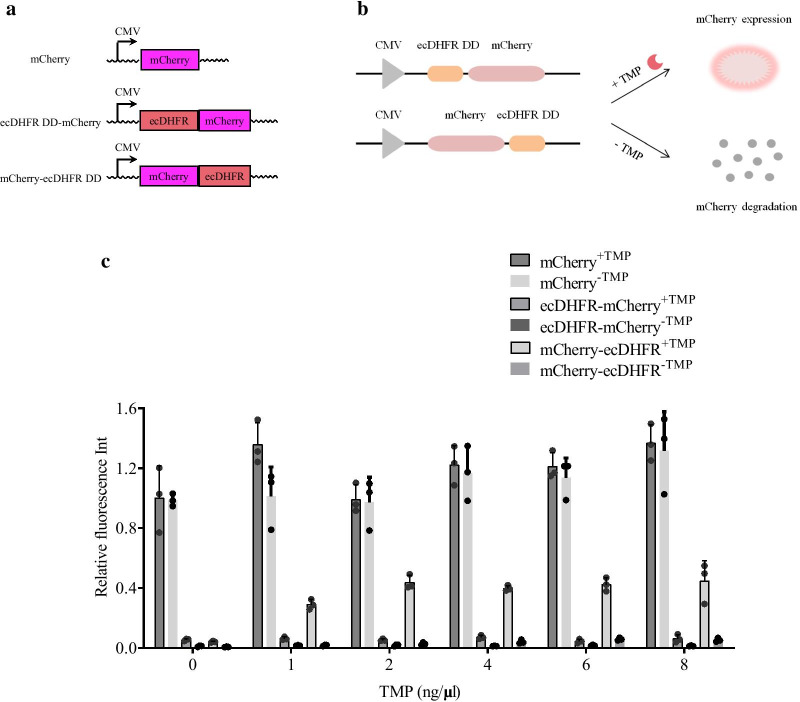
Detection of ecDHFR DD-fused mCherry expression induced by TMP. **a** Schematic representation of the engineered ecDHFR DD-fused *mCherry* expression plasmids. ecDHFR DD was fused to the C- or N-terminal of *mCherry*. **b** Illustration of the mechanism by which conditionally destabilized ecDHFR DD regulates mCherry expression. mCherry gene is fused with the ecDHFR DD, which triggered its rapid degradation. The TMP prevents the destabilization and degradation of mCherry. **c** Red optical density statistics of mCherry expression in mCherry-, mCherry-ecDHFR DD-, and ecDHFR DD-mCherry-transfected 293T cells in the presence of TMP (0, 1, 2, 4, 6, or 8 ng/μL) for 24 h. mCherry integrated density was detected at 24 h post-TMP removal

ecDHFR DD can regulate the expression of the fused proteins with the effect of TMP [4]. To assess the versatility of the ecDHFR DD system, two ecDHFR DD-regulated fluorescence reporters, namely mCherry-ecDHFR DD and ecDHFR DD-mCherry, were designed and ecDHFR DD was fused at the C- and N-terminal of mCherry (Fig. 1a). Three constructs, including mCherry, mCherry-ecDHFR DD, and ecDHFR DD-mCherry, were used to analyze the mCherry expression under the control of the ecDHFR DD-TMP combination. TMP was expected to prevent the destabilizing effect of ecDHFR DD, thereby protecting mCherry from degradation (Fig. 1b). The three fluorescence reporters were transfected into 293T cells, and different doses of TMP (0, 1, 2, 4, 6, and 8 ng/μL) were added 24 h post-transfection. At 48 h post-transfection, lower mCherry expression was observed in mCherry-ecDHFR DD- and ecDHFR DD-mCherry-transfected cells without TMP treatment compared with the mCherry-transfected group, whereas it was significantly increased in mCherry-ecDHFR DD-transfected cells upon TMP treatment, reaching the maximum mCherry effective concentration at 2 ng/μL TMP (Fig. 1c and Additional file 1: Fig. S1). Therefore, the optimal concentration of 2 ng/μL TMP was selected for the following experiments. Of note, the inhibitory effect could be restored after TMP removal for 48 h. However, the mCherry expression did not recover after the TMP treatment in ecDHFR-mCherry-transfected cells, suggesting that the N-terminal ecDHFR DD fusion could be insensitive to all TMP concentrations (Fig. 1c and Additional file 1: Fig. S1). These results showed that mCherry expression could be effectively regulated by the ecDHFR DD-TMP combination, under the control of the ecDHFR DD fused at the C-terminal of *mCherry*.

### ecDHFR DD well-regulated ecRESCUE system expression

**Fig. 2:**
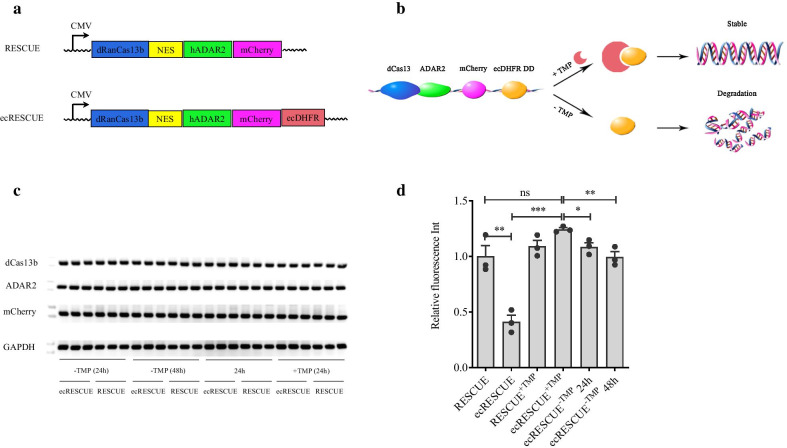
Detection of ecDHFR DD-fused RESCUE system expression induced by TMP. **a** Schematic representation of the engineered ecDHFR DD-fused RESCUE expression plasmids. ecDHFR DD was fused to the C-terminal of mCherr*y*. **b** Illustration of the mechanism by which conditionally destabilized ecDHFR DD regulates dRanCas13b, ADAR2, and mCherry expression. ADAR2 and mCherry are fused with the ecDHFR DD. The TMP prevents the destabilization and degradation of dRanCas13b, ADAR2, and mCherry. **c** *dRanCas13b*, *ADAR2*, and mCherry expression in RESCUE- and ecRESCUE-transfected 293T cells with or without TMP. Without TMP (24 h): at 48 h post-transfection without TMP; + TMP: at 24 h post-TMP treatment; +  − TMP (24 h): at 24 h post-TMP removal; +  − TMP (48 h): at 48 h post-TMP removal. **d** Red optical density statistics of mCherry in RESCUE- and ecRESCUE-transfected 293T cells. RESCUE and ecRESCUE: at 48 h post-transfection without TMP; + TMP: at 24 h post-TMP treatment; − TMP (24 h): at 24 h post-TMP removal; − TMP (48 h): at 48 h post-TMP removal. Values are shown as mean ± SEM (n = 3). Different superscript letters indicate significant differences as follows: *P < 0.05; **P < 0.01; ***P < 0.001; ns indicate there is no significance

Next, based on above-described results, the ability of ecDHFR DD to regulate the expression of the two main functional elements of RESCUE RNA editor (dRanCas13b and human ADAR2), which reduced the RESCUE effective time and off-target events, was evaluated (Fig. 2b). Two mCherry expression RNA base editors were used; RESCUE-mCherry and RESCUE-mCherry-ecDHFR RNA base editors, with mCherry and ecDHFR DD fused at the C-terminal of the RESCUE and RESCUE-mCherry, respectively, were considered for further experiments (Fig. 2a). The two RNA base editors, RESCUE and ecRESCUE, were transfected into 293T cells, 2 ng/μL TMP was added to the cells 24 h post-transfection, and fluorescence analysis was performed to detect the expression of dRanCas13b and ADAR2. The results showed that mCherry levels were markedly decreased in ecRESCUE-transfected cells without TMP treatment compared to the RESCUE-transfected group. Moreover, 2 ng/μL TMP treatment could inhibit the ecDHFR DD-mediated fused protein degradation and partially recover the expression of the RESCUE-mCherry protein. After TMP was removed for 24 and 48 h, the inhibitory effect of ecDHFR DD was markedly restored and the inhibitory effect was remarkably increased with the extension of TMP removal time (Fig. 2d and Additional file 1: Fig. S2). To confirm that ecDHFR DD regulated protein levels at the post-translational level without affecting mRNA expression, the mRNA expression of *dRanCas13b*, *ADAR2*, and fused mCherry in both the RESCUE and ecRESCUE RNA base editors was evaluated by performing reverse transcriptase-polymerase chain reaction (RT-PCR). As expected, the expression of *dRanCas13b*, *ADAR2*, and fused mCherry in the presence or absence of TMP was similar in the two RNA base editor systems (Fig. 2c). Taken together, these data demonstrate that C-terminal ecDHFR DD fusion enables remarkable, rapid, and reversible control of the expression of the RESCUE-related functional proteins, thereby making ecRESCUE a suitable tool for the generation of inducible RNA editing systems.

### ecRESCUE mediates A-to-I and C-to-U base editing of endogenous mRNA

**Fig. 3:**
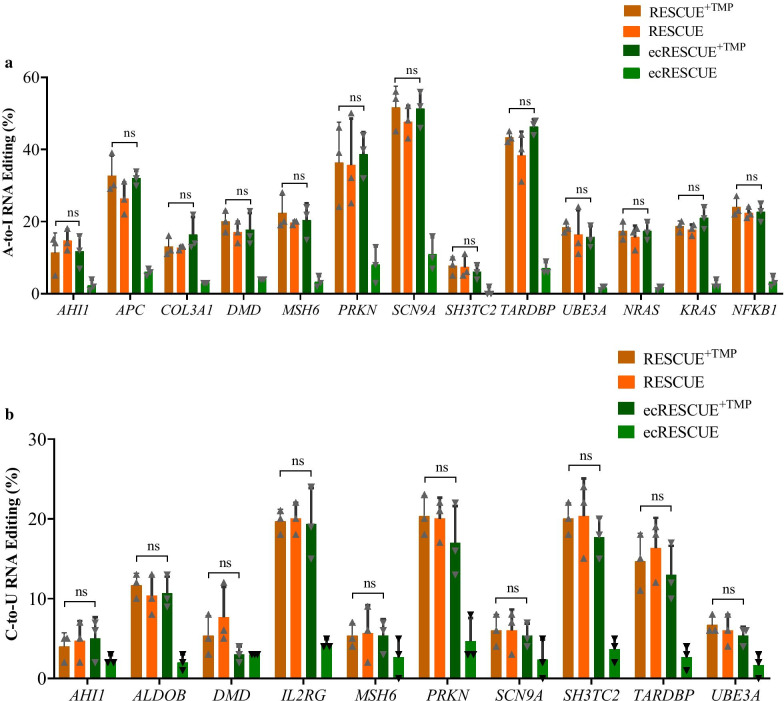
ecDHFR DD-fused RESCUE system mediates endogenous mRNA A-to-I and C-to-U editing. RESCUE and ecRESCUE system mediated endogenous RNA **a** A-to-I and **b** C-to-U base editing in 293T cells. + TMP: 2 ng/μL TMP was added after 24 h of transfection. Values are shown as mean ± SEM (n = 3). ns, no significance

Furthermore, the endogenous RNA editing efficiency of ecRESCUE was evaluated in 293T cells. To detect A-to-I and C-to-U editing efficiency, 13 endogenous sites were selected. Specifically, we considered *KRAS*, *NRAS*, *NFKB1*, *AHI1*, *APC*, *COL3A1*, *DMD*, *MSH6*, *PRKN*, *SCN9A*, *SH3TC2*, *TARDBP*, and *UBE3A* for performing A-to-I editing, and *KRAS*, *NRAS*, *NFKB1*, *AHI1*, *ALDOB*, *DMD*, *IL2RG*, *MSH6*, *PRKN*, *SCN9A*, *SH3TC2*, *TARDBP*, and *UBE3A* for performing C-to-U editing. For each endogenous site, one single guide RNA (sgRNA, Additional file 1: Table S1) fused with GFP was designed to guide the precise endogenous RNA editing. To compare the RNA editing efficiency between the RESCUE and ecRESCUE systems, two experiment groups were set up, including RESCUE/ecRESCUE group and RESCUE/ecRESCUE + TMP group, in which 2 ng/μL TMP was added at 24 h post-transfection. At 48 h post-transfection, 2 × 10^4^ GFP-positive cells were collected by fluorescence-activated cell sorting, and total RNA from these cells was collected. The target sequences of the endogenous sites were amplified and subjected to Sanger sequencing. The mRNA base editing efficiency of these targets were calculated by using the Edit R web tool (https://moriaritylab.shinyapps.io/editr_v10/). [6] The results showed relatively less RNA editing efficiency with the ecRESCUE system in the absence of TMP. Moreover, no significant differences were observed in both A-to-I (Fig. 3a) and C-to-U (Fig. 3b) RNA editing efficiency between the RESCUE and ecRESCUE RNA editors with TMP treatment, demonstrating that the ecRESCUE RNA editor was a versatile RNA base editor.

### Transcriptome-wide A-to-I and C-to-U RNA off-targets were significantly decreased in ecRESCUE system

**Fig. 4:**
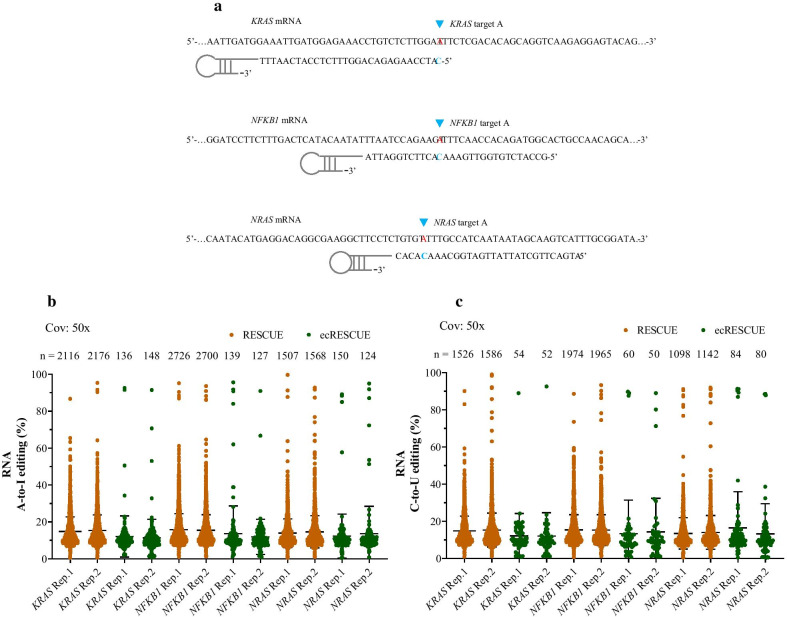
A-to-I and C-to-U off-target events of RESCUE and ecDHFR DD-fused RESCUE RNA editors. **a** Schematic of *KRAS*, *NFKB1*, and *NRAS* target sites and guide RNA design. Manhattan plots showing the distribution of modified **b** adenines and **c** cytosines across the transcriptome for *KRAS*, *NFKB1*, and *NRAS* RNA A-to-I and C-to-U editing, respectively, by RESCUE and ecRESCUE editors (n = 2 for each system; coverage: × 50). n, total number of modified adenines identified

We evaluated whether the new RNA base editor could decrease the off-target effects. To this end, three endogenous sites (*KRAS*, *NFKB1*, and *NRAS*) were examined for mRNA A-to-I base editing by using the RESCUE and ecRESCUE RNA editors (Fig. 4a). In a first approach, transcriptome-wide off-targets were assessed by performing RNA-sequencing of all mRNA samples. The results showed that there were 2116 and 2176 A-to-I off-targets in *KRAS* targeting cells obtained by using RESCUE, and 136 and 148 off-targets were obtained by using ecRESCUE with 50 × coverage; 2726 and 2700 A-to-I off-targets in *NFKB1* targeting cells were obtained by using RESCUE, and 139 and 127 off-targets were obtained by using ecRESCUE with 50 × coverage; and 1507 and 1568 A-to-I off-targets in *NRAS* targeting cells were obtained by using RESCUE, and 150 and 124 off-targets were obtained by using ecRESCUE with 50 × coverage (Fig. 4b). These results showed that the ecRESCUE RNA editor led to the formation of markedly less A-to-I off-targets than the standard RESCUE system. Furthermore, the RNA-sequencing results also showed that there were 1,526 and 1,586 C-to-U off-targets in *KRAS* targeting cells obtained by using RESCUE, and 54 and 52 off-targets were obtained by using ecRESCUE with 50 × coverage; 1,974 and 1,965 C-to-U off-targets in *NFKB1* targeting cells were obtained by using RESCUE, and 60 and 50 off-targets were obtained by using ecRESCUE with 50 × coverage; and 1,098 and 1,142 C-to-U off-targets in *NFKB1* targeting cells were obtained by using RESCUE, and 84 and 80 off-targets were obtained by using ecRESCUE with 50 × coverage (Fig. 4c); demonstrating that the ecRESCUE RNA editor led to the formation of markedly less C-to-U off-targets. Taken together, these results demonstrated that the ecRESCUE editor led to the formation of remarkably less off-targets, thus considerably improving the RNA editor system specificity.

## Discussion

RNA base editing technologies result in temporary changes in the cell state or modify the function of proteins by mRNA single base inversions without inflicting damage to the genome, which holds considerable advantage in the treatment of certain genetic disorders. Currently, there are two main efficient RNA base editors, namely REPAIR [1] and RESCUE [2]. The REPAIR RNA editor can be used to achieve precise A-to-I editing by performing fusion of dRanCas13b with ADAR2, whereas the RESCUE system can be used to precisely achieve both A-to-I and C-to-U RNA edits by performing fusion of dRanCas13b with the revolutionized ADAR2. However, both systems result in A-to-I and C-to-U off-target edits. SNPs can alter off-target sites by increasing or decreasing the number of mismatches between a genomic region and the gRNA sequence. Also, these off-target SNPs may directly affect the expression pattern of different RNA, such as mRNA, miRNA and circRNA. The A > I editing, mediated by ADAR2, is an antagonist of circRNA (Circular RNA) production. circRNA can regulate gene expression by influencing the transcription, the mRNA turnover, and translation by sponging RNA-binding proteins and miRNAs [7]. Off-target SNPs may affect the networks among mRNA, miRNA, circRNA and other RNAs.

When considering applicability in the treatment of genetic diseases, it is important to assess whether the RNA editing tools are indeed safe. Despite the numerous RNA off-target effects produced by RNA base editors, the off-target events have been significantly decreased using the RESCUE system compared with the REPAIR RNA editor. Nonetheless, the results presented herein demonstrated that it was feasible to reduce the incidence of off-target SNPs with the utilization of the RESCUE editor by applying a TMP conditionally induced ecDHFR DD system. This study showed that ecDHFR DD-TMP combination could effectively regulate the expression of mCherry when fused with the C-terminal mCherry sequence. Moreover, this system also demonstrated the regulation of the dRanCas13b and ADAR2 levels with reduction in the off-target incidence compared with the RESCUE system. As expected, ecDHFR DD markedly inhibited the expression of mCherry fused with the C-terminal of the *ADAR2* gene and remarkably reduced the number of off-target SNPs without influencing the endogenous RNA editing efficiency. Hence, ecRESCUE represents a novel approach to reduce RNA off-target editing events with similar efficiency but higher specificity than that observed with the application of the RESCUE editing system. To a certain extent, it may represent a better approach to achieve RNA editing, or even DNA editing, by regulating heterologous protein expression.

Despite the enhanced outcome, the ecRESCUE system is associated with the induction of off-target events. Previous study showed that without the effect of the TMP, ecDHFR DD exhibit a functional background, which may not regulate the fused protein degradation completely. Particularly, they investigated a double architecture for the DD-gene-DD-fused sequence and found that such a construction completely suppressed the protein function in an uninduced state [8]. Hence, it is possible to improve the ecRESCUE system to present with less off-targets via application of a double architecture for DD-RESCUE-DD.

## Conclusions

In summary, this study describes the new ecRESCUE RNA editor that can be utilized for efficient and specific on-target RNA base editing, thereby representing a safe tool for conducting RNA base editing and genetic function research.

## Supplementary Information

**Additional file 1. Figure S1**. TMP induced ecDHFR DD-regulated mCherry protein expression.** Figure S2**. Detection of expression of TMP-induced ecDHFR DD fused RESCUE-mCherry protein.** Table S1**. Guide sequences used for endogenous gene editing.** Table S2**. Primer sequences used for PCR fragments amplification.** Table S3**. Primer sequences used for dCas13b, ADAR2 and mCherry mRNA expression detection by RT-PCR.** Table S4**. mCherry reporter expression vectors.** Table S5**. RNA base editor expression vectors.

## Data Availability

The RNA-seq data are deposited at the NCBI Bioproject (PRJNA656893).

## Abbreviations

REPAIR: RNA editing for programmable A-to-I replacement
RESCUE: RNA editing for specific C-to-U exchange
ecRESCUE: EcDHFR DD-regulated RNA editing for specific C-to-U exchange
A-to-I: adenosine-to-inosine
C-to-U: cytidine-to-uridine
ADAR2: adenosine deaminase acting on RNA type 2
FACS: fluorescence activating cell sorter
