# Supplementary material for: ecRESCUE: a novel ecDHFR-regulated RESCUE system with reduced RNA off-targeting activity
Source: Cell Commun Signal. 2021 Jul 31;19:81. doi: 10.1186/s12964-021-00759-2 (PMC8325194; doi:10.1186/s12964-021-00759-2)
Supplement: Supplementary file 2 — Additional file 1. Figure S1. TMP induced ecDHFR DD-regulated mCherry protein expression. Figure S2. Detection of expression of TMP-induced ecDHFR DD fused RESCUE-mCherry protein. Table S1. Guide sequences used for endogenous gene editing. Table S2. Primer sequences used for PCR fragments amplification. Table S3. Primer sequences used for dCas13b, ADAR2 and mCherry mRNA expression detection by RT-PCR. Table S4. mCherry reporter expression vectors. Table S5. RNA base editor expression vectors. [file 12964_2021_759_MOESM2_ESM.pdf]

1. Supplemental Figures

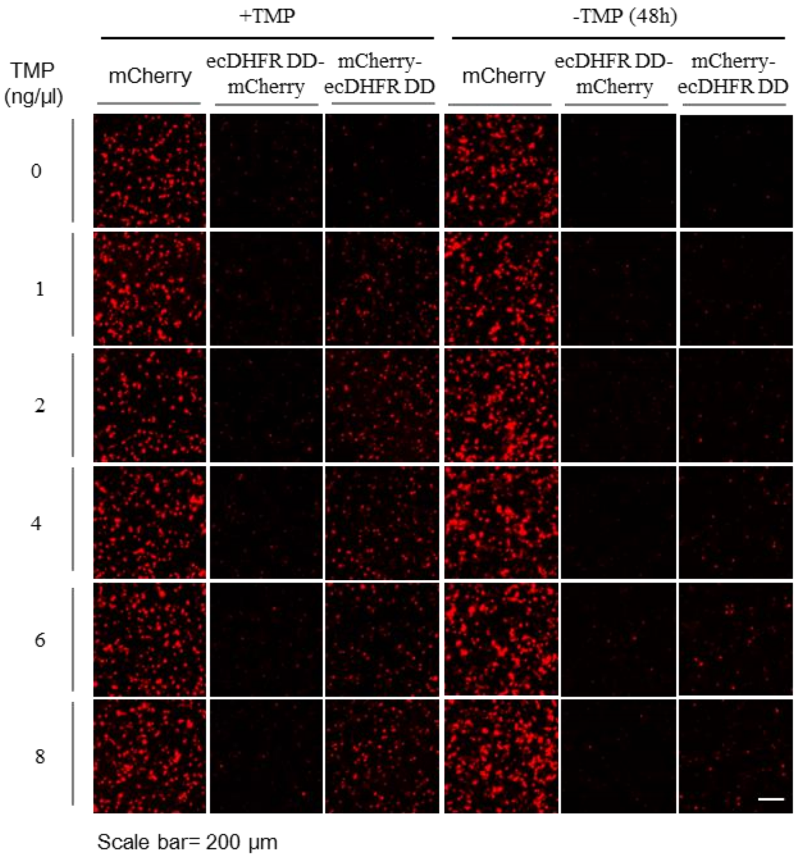

Figure S1 TMP induced ecDHFR DD-regulated mCherry protein expression.

At 24 h post-transfection, 0, 2, 4, 6 and 8 ng/µL TMP was added into mCherry, ecDHFR DD-mCherry and mCherry-ecDHFR DD expressed 293T cells to induce the ecDHFR DD fused mCherry protein expression. +TMP: at 24 h post TMP treatment; -TMP (48h): at 48 h post TMP removing. Scales bars, 200 µm.

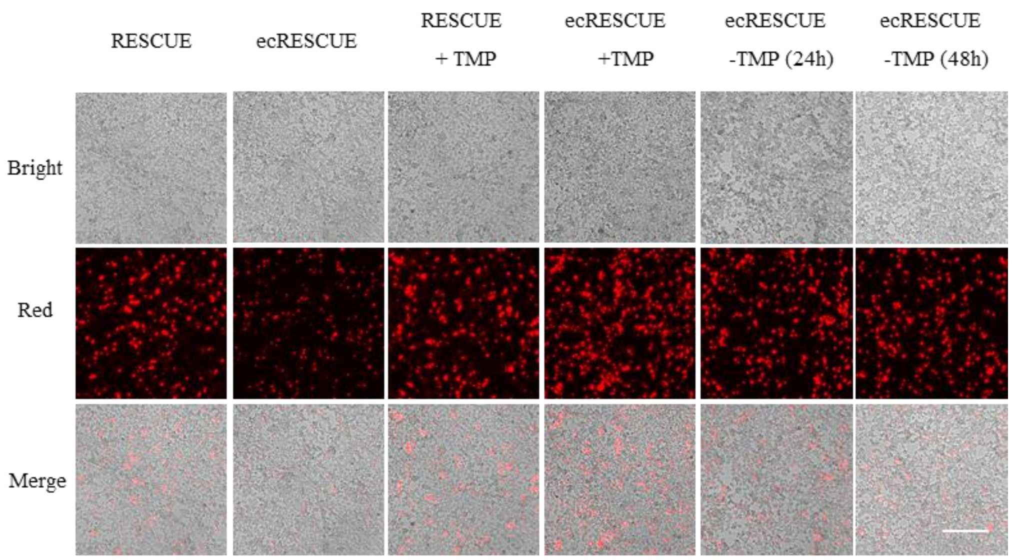

Figure S2 Detection of expression of TMP-induced ecDHFR DD fused RESCUE-mCherry protein.

At 24 h post-transfection, 2 ng/μL TMP was added into RESCUE and ecRESCUE expressed 293T cells to induce ecDHFR DD fused protein expression. Scales bars, 200 μm.

2. Table S1 Guide sequences used for endogenous gene editing.

Targeted gene	Motif	Base flip/position	Spacer sequence
AHI1	GAU	A/24	TATGAAAGAAACTACAAGTGATGATCCCGA
AHI1	GCU	C/22	CAAGTGATGATGTAAGTGCTGCTAACACTA
ALDOB	GAU	A/22	AAACCACCATTCAAGGGCTTGATGGCCTCT
ALDOB	GCU	C/23	GATGGCCTCTCAGAGCGCTGTGCTCAGTAC
APC	UAA	A/22	GAGCTTAACTTAGATAGCAGTAATTTCCCT
APC	UCC	C/24	AGTAAAACTGCGGTCAAAAATGTCCCTCCG

BMPR2	GAU	A/19	CTTTTCTTTGCCCTCCTGATTCTTGGCTGG
BMPR2	UCG	C/21	TGGCCCAGGGATGACTTCCTCGCTGCAGCG
COL3A1	UAU	A/24	CTGGGAGAAATGGTGACCCTGGTATTCCAG
COL3A1	UCG	C/22	TGGAGTAGCAGTAGGAGGACTCGCAGGCTA
DMD	UAU	A/23	CAACGTCCCTCTCTGCGTGGATATGTGTCT
DMD	GCU	C/22	GATATGTGTCTGAACTGGCTGCTGAATGTT
IL2RG	GAU	A/24	TGCTAAAACTGCAGAATCTGGTGATCCCCT
IL2RG	GCA	C/21	ACTGTTTGGAGCACTTGGTGCAGTACCGGA
MSH6	UAG	A/21	CACATATATCCAAGTATGATAGAGTGGTGA
MSH6	UCU	C/20	GAGTGGTGAGGAGGGAGATCTGTAGGATCA
PRKN	UAC	A/22	GTCCAGCAGGTAGATCAATCTACAACAGCT
PRKN	UCU	C/21	CTGTGGGGCTGGCTGTCATTCTGCACACTG
SCN9A	UAU	A/23	CAATGCCACACCTGCTTTATATATGCTTTC
SCN9A	UCU	C/24	TTTCAGTCCTCTAAGAAGAATATCTATTAA
SH3TC2	UAC	A/23	TCCACCTACCTTAATTTAGGCTACGTATCC
SH3TC2	UCU	C/21	ATGTGTCTAGAACATCTCCTCTTTGACCAC
TARDBP	UAG	A/23	TAATGTCACAGCGACATATGATAGATGGAC
TARDBP	UCU	C/23	TATGGTGCAGGTCAAGAAAGATCTTAAGAC
UBE3A	UAU	A/25	GCTTCCTGTCCAACTTTTCTTCGTATGGAT
UBE3A	UCU	C/24	CTTTATAAGATTAATGCAAAACTCTGTGAT
KRAS	UAU	A/25	TTCAGAATCATTTTGTGGACGAATATGATC
KRAS	ACG	C/20	TTCAGAATCATTTTGTGGACGAATATGATC

NFKB1	UAU	A/13	TAATCCAGAAGTATTTCAACCACAGATGGC
NFKB1	UCA	C/17	TAATCCAGAAGTATTTCAACCACAGATGGC
NRAS	UAU	A/26	GGACAGGCGAAGGCTTCCTCTGTGTATTTG

3. Primers used in this study.

Table S2. Primer sequences used for PCR fragments amplification.

Targeted gene	PCR primer sequences (5'-3')
AHI1	F: GCTGAGAGATGCCTACAGCT; R: CTGGCTGTGGCTTTGTATGT
ALDOB	F: GCCGGCAGTTCCGAGAAATC; R: GTAGACAGCAGCCAGGACCT
APC	F: GCAAGTTGAGGCACTGAAGA; R: CAGCAAGAAGCAATGACCTC
BMPR2	F: GGAATTTCTGCAGCGGCATG; R: GCCATAGCAGGTGCTACCTT
COL3A1	F: CTCAGGATCCGTTCTCTGCG; R: TCCTGGAGGGCCTGAAGGAC
DMD	F: CTCCGAAGACTGCAGAAGGC; R: TACTGCCCCCAAAGGATGCA
IL2RG	F: AGCCTACCAACCTCACTCTG; R: TTCACTCCAATGCTGAGCAC
MSH6	F: AGCTGTACCACATGGATGCT; R: GGGGGATAGTGTGCCACTAG
PRKN	F: GTGACCTGGATCAGCAGAGC; R: CACTAGTCCCAGGGCAGTGT
SCN9A	F: CCCCAAAGCCAAGCAGTGAC; R: CAGTTCCACGGGTCACGAAG
SH3TC2	F: ACCCCTACAGGAAGCTGCTC; R: TCCAACTCGGAGCCAGCTTC
TARDBP	F: ATGCTGGCTGGGGAAATCTG; R: CAAAGGCCCTGAATGGCTTG
UBE3A	F: GGCGACGACAGATCAGGAGA; R: CTTTCCGGAAGCTCTGTACC
KRAS	F: GGCTCGGCCAGTACTCCCGG; R: GTCCTCATGTACTGGTCCCTCA
NFKB1	F: TTAGGAGGGAGAGCCCACCC; R: AGTGCAGATCCCATCCTCAC

Table S3. Primer sequences used for dCas13b, ADAR2 and mCherry mRNA expression detection by RT-PCR.

Targeted gene	PCR primer sequences (5’-3’)
dCas13b	F: GCACTACGAGTATTGGCAGC; R: GTACAGCCGCAGCTTCTTCT
ADAR2	F: CCTGTGGAGATGCCAGAATC; R: CATTGTTGCGCACTGGAATC
mCherry	F: TCCTGTCCCCTCAGTTCATG; R: GCCGTCCTCGAAGTTCATCA
GAPDH	F: AGAAGGCTGGGGCTCATTTG; R: AGGGGCCATCCACAGTCTTC

Table S4. mCherry reporter expression vectors

ecDHFR DD, and mCherry are highlighted in blue, and red, respectively. The start and stop codons are capitalized.

**mCherry**	ATGgtgagcaagggcgaggaggataacatggccatcatcaaggagttcatgcgcttcaaggtgcacatggag<br>ggctccgtgaacggccacgagttcgagatcgagggcgagggcgagggccgcccctacgagggcacccagac<br>cgccaagctgaaggtgaccaagggtggcccccctgcccttcgcctgggacatcctgtcccctcagttcatgtacgg<br>ctccaaggcctacgtgaagcaccccgccgacatccccgactacttgaagctgtccttccccgagggcttcaagtg<br>ggagcgcgtgatgaacttcgaggacggcggcgtggtgaccgtgacccaggactcctccctgcaggacggcga<br>gttcatctacaaggtgaagctgcgcggcaccaacttcccctccgacggccccgtaatgcagaagaagaccatgg<br>gctgggaggcctcctccgagcggatgtaccccgaggacggcgccctgaagggcgagatcaagcagaggctga<br>agctgaaggacggcggccactacgacgctgaggtcaagaccacctacaaggccaagaagcccgtgcagctgc<br>ccggcgcctacaacgtcaacatcaagttggacatcacctcccacaacgaggactacaccatcgtggaacagtac<br>gaacgcgccgagggccgccactccaccggcggcatggacgagctgtacaagTAA

**mCherry-**<br>**ecDHFR DD**	ATGgtgagcaagggcgaggaggataacatggccatcatcaaggagttcatgcgcttcaaggtgcacatggag<br>ggctccgtgaacggccacgagttcgagatcgagggcgagggcgagggccgcccctacgagggcacccagac<br>cgccaagctgaaggtgaccaagggtggccccctgcccttcgcctgggacatcctgtcccctcagttcatgtacgg<br>ctccaaggcctacgtgaagcaccccgccgacatccccgactacttgaagctgtccttccccgagggcttcaagtg<br>ggagcgcgtgatgaacttcgaggacggcggcgtggtgaccgtgacccaggactcctccctgcaggacggcga<br>gttcatctacaaggtgaagctgcgcggcaccaacttcccctccgacggccccgtaatgcagaagaagaccatgg<br>gctgggaggcctcctccgagcggatgtaccccgaggacggcgccctgaagggcgagatcaagcagaggctga<br>agctgaaggacggcggccactacgacgctgaggtcaagaccacctacaaggccaagaagcccgtgcagctgc<br>ccggcgcctacaacgtcaacatcaagttggacatcacctcccacaacgaggactacaccatcgtggaacagtac<br>gaacgcgccgagggccgccactccaccggcggcatggacgagctgtacaagatcagtctgattgcggcgttag<br>cggtagattacgttatcggcatggaaaacgccatgccgtggaacctgcctgccgatctcgcctggtttaaacgcaa<br>caccttaaataaacccgtgattatgggccgccatacctgggaatcaatcggtcgtccgttgccaggacgcaaaaat<br>attatcctcagcagtcaaccgagtacggacgatcgcgtaacgtgggtgaagtcggtggatgaagccatcgcggc<br>gtgtggtgacgtaccagaaatcatggtgattggcggcggtcgcgttattgaacagttcttgccaaaagcgcaaaaa<br>ctgtatctgacgcatatcgacgcagaagtggaaggcgacacccatttcccggattacgagccggatgactgggaa<br>tcggtattcagcgaattccacgatgctgatgcgcagaactctcacagctattgctttgagattctggagcggTAA
**ecDHFR**<br>**DD-mCherry**	ATGatcagtctgattgcggcgttagcggtagattacgttatcggcatggaaaacgccatgccgtggaacctgcct<br>gccgatctcgcctggtttaaacgcaacaccttaaataaacccgtgattatgggccgccatacctgggaatcaatcg<br>gtcgtccgttgccaggacgcaaaaatattatcctcagcagtcaaccgagtacggacgatcgcgtaacgtgggtga<br>agtcggtggatgaagccatcgcggcgtgtggtgacgtaccagaaatcatggtgattggcggcggtcgcgttattg<br>aacagttcttgccaaaagcgcaaaaactgtatctgacgcatatcgacgcagaagtggaaggcgacacccatttcc<br>cggattacgagccggatgactgggaatcggtattcagcgaattccacgatgctgatgcgcagaactctcacagcta

	ttgctttgagattctggagcggg gtgagcaagggcgaggaggataacatggccatcatcaaggagttcatgcgcttc<br>aaggtgcacatggagggctccgtgaacggccacgagttcgagatcgagggcgagggcgagggccgcccctac<br>gagggcacccagaccgccaagctgaaggtgaccaagggtggccccctgcccttcgcctgggacatcctgtccc<br>ctcagttcatgtacggctccaaggcctacgtgaagcaccccgccgacatccccgactacttgaagctgtccttccc<br>cgagggcttcaagtgggagcgcgtgatgaacttcgaggacggcggcgtggtgaccgtgacccaggactcctcc<br>ctgcaggacggcgagttcatctacaaggtgaagctgcgcggcaccaacttcccctccgacggccccgtaatgca<br>gaagaagaccatgggctgggaggcctcctccgagcggatgtaccccgaggacggcgccctgaagggcgagat<br>caagcagaggctgaagctgaaggacggcggccactacgacgctgaggtcaagaccacctacaaggccaagaa<br>gcccgtgcagctgcccggcgcctacaacgtcaacatcaagttggacatcacctcccacaacgaggactacacca<br>tcgtggaacagtacgaacgcgccgagggccgccactccaccggcggcatggacgagctgtacaagTAA

Table S5. RNA base editor expression vectors

ecDHFR DD, dCas13, ADAR2 and mCherry are highlighted in blue, yellow, green and red, respectively. The start and stop codons are capitalized.

**RESCUE**	ATGgagaagcccctgctgcctaacgtgtataccctgaagcacaagttcttttggggcgccttcctgaatatcgcc<br>cggcacaacgcctttatcaccatctgccacatcaatgagcagctgggcctgaaaacccccagcaacgacgataa<br>gatcgtggacgtggtgtgcgagacatggaacaatatcctgaacaatgaccacgatctgctgaagaagtcccagct<br>gaccgagctgatcctgaagcacttcccttttctgacagccatgtgctaccaccccctaagaaggagggcaagaa<br>gaagggccaccagaaggagcagcagaaggagaaggagagcgaggcacagtcccaggcagaggccctgaac<br>ccatccaagctgatcgaggccctggagatcctggtgaatcagctgcactctctgGCAaactactatagcGCCt<br>ataagcacaagaagcccgacgccgagaaggatatcttcaagcacctgtataaggccttcgacgcctctctgagaa<br>tggtgaaggaggattataaggcccacttcaccgtgaatctgacaagggactttgcccacctgaaccgcaagggca

	agaacaagcaggacaatcccgacttcaacagatacagattcgagaaggatggcttctttaccgagtctggcctgct<br>gttctttacaaatctgtttctggacaagagggatgcctattggatgctgaagaaggtgtccggcttcaaggcctctca<br>caagcagcgcgagaagatgaccacagaggtgttttgcaggagccgcatcctgctgcccaagctgaggctggagt<br>cccgctacgaccacaaccagatgctgctggatatgctgtctgagctgagcagatgtcctaagctgctgtatgagaa<br>gctgagcgaggagaataagaagcacttccaggtggaggccgacggctttctggatgagatcgaggaggagcag<br>aacccattcaaggacaccctgatccggcaccaggatagattcccctactttgccctgaggtatctggacctgaatga<br>gtccttcaagtctatccgctttcaggtggatctgggcacataccactattgtatctacgacaagaagatcggcgatga<br>gcaggagaagaggcacctgacccgcacactgctgtccttcggccggctgcaggactttaccgagatcaacagac<br>cccaggagtggaaggccctgaccaaggacctggattacaaggagacatccaatcagcctttcatctctaagacca<br>caccacactatcacatcaccgacaacaagatcggctttcggctgggcacaagcaaggagctgtacccctccctgg<br>agatcaaggatggcgccaatagaatcgccaagtacccttataactccggcttcgtggcccacgcctttatctctgtg<br>cacgagctgctgcctctgatgttctaccagcacctgaccggcaagagcgaggacctgctgaaggagacagtgcg<br>gcacatccagagaatctataaggacttcgaggaggagcggatcaataccatcgaggatctggagaaggcaaacc<br>agggcagactgccactgggagcctttcccaagcagatgctgggcctgctgcagaataagcagcctgatctgtccg<br>agaaggccaagatcaagatcgagaagctgatcgccgagacaaagctgctgtctcacaggctgaacacaaagct<br>gaagagctccccaaagctgggcaagcggagagagaagctgatcaagacaggcgtgctggccgactggctggt<br>gaaggatttcatgcgctttcagcccgtggcctacgacgcccagaatcagcctatcaagtctagcaaggccaactcc<br>accgagttctggtttatcaggcgcgccctggccctgtacggaggagagaagaataggctggagggctatttcaag<br>cagaccaacctgatcggcaacacaaatccacaccccttcctgaacaagtttaattggaaggcctgcaggaatctgg<br>tggatttctaccagcagtatctggagcagcgcgagaagtttctggaggccatcaagaaccagccatgggagccct<br>accagtattgcctgctgctgaagatccctaaggagaacaggaagaatctggtgaagggatgggagcagggagg<br>aatctctctgccacggggcctgtttaccgaggccatcagagagacactgtctgaggacctgatgctgagcaagcc

	aatccgcaaggagatcaagaagcacggccgggtgggcttcatcagcagagccatcaccctgtactttaaggaga<br>agtatcaggataagcaccagagcttctacaatctgtcctataagctggaggcaaaggcaccactgctgaagcggg<br>aggagcactacgagtattggcagcagaacaagcctcagtctccaaccgagagccagaggctggagctgcacac<br>aagcgaccgctggaaggattacctgctgtataagagatggcagcacctggagaagaagctgcggctgtaccgga<br>atcaggacgtgatgctgtggctgatgaccctggagctgacaaagaaccacttcaaggagctgaacctgaattatca<br>ccagctgaagctggagaatctggccgtgaacgtgcaggaggccgatgccaagctgaaccctctgaatcagaccc<br>tgcccatggtgctgcctgtgaaggtgtaccctgccacagcctttggcgaggtgcagtaccacaagaccccaatca<br>ggacagtgtatatccgcgaggagcacaccaaggccctgaagatgggcaacttcaaggccctggtgaaggaccg<br>gagactgaatggcctgttctcttttatcaaggaggagaacgatacacagaagcacccaatcagccagctgcggct<br>gaggcgcgagctggagatctaccagtctctgagagtggacgccttcaaggagacactgagcctggaggagaag<br>ctgctgaataagcacacatccctgtcctctctggagaacgagtttagggccctgctggaggagtggaagaaggag<br>tatgccgccagctccatggtgacagacgagcacatcgccttcatcgccagcgtgGCcaatgccttctgcGCca<br>accagtaccccttctacaaggaggccctgcacgcccctatcccactgttcaccgtggcccagcccaccacagag<br>gagaaggatggcctgggaatcgcagaggccctgctgaaggtgctgagggagtactgtgagatcgtgaagtccc<br>agatcggatcccttcaactgcctccacttgaaagactgacactgggatcccagctgcatttaccgcaggttttagctg<br>acgctgtctcacgcctggtcataggtaagtttggtgacctgaccgacaacttctcctcccctcacgctcgcagaata<br>ggtctggctggagtcgtcatgacaacaggcacagatgttaaagatgccaaggtgatatgtgtttctacaggagcaa<br>aatgtattaatggtgaatacctaagtgatcgtggccttgcattaaatgactgccatgcagaaatagtatctcggagatc<br>cttgctcagatttctttatacacaacttgagctttacttaaataacgaggatgatcaaaaaagatccatctttcagaaatc<br>agagcgaggggggtttaggctgaaggagaatatacagtttcatctgtacatcagcacctctccctgtggagatgcc<br>agaatcttctcaccacatgaggcaatcctggaagaaccagcagatagacacccaaatcgtaaagcaagaggaca<br>gctacggaccaaaatagaggctggtcaggggacgattccagtgcgcaacaatgcgagcatccaaacgtgggac

	ggggtgctgcaaggggagcggctgctcaccatgtcctgcagtgacaagattgcacgctggaacgtggtgggcatccagggatcactgctcagcattttcgtggagcccatttacttctcgagcatcatcctgggcagcctttaccacggggaccacctttccagggccatgtaccagcggatctccaacatagaggacctgccacctctctacaccctcaacaagcctttgctcacaggcatcagcaatgcagaagcacggcagccagggaaggcccccatattcagtgtcaactggacggtaggcgactccgctattgaggtcatcaacgccacgactgggaagggagagctgggccgcgcgtcccgcctgtgtaagcacgcgttgtactgtcgctggatgcgtgtgcacggcaaggttccctcccacttactacgctccaagattaccaagcccaacgtgtaccatgagacaaagctggcggcaaaggagtaccaggccgccaaggcgcgtctgttcacagccttcatcaaggcggggctgggggcctgggtggagaagcccaccgagcaggaccagttctcactcacgcttcaactgcctccacttgaaagactgacactggtgagcaagggcgaggaggataacatggccatcatcaaggagttcatgcgcttcaaggtgcacatggagggctccgtgaacggccacgagttcgagatcgagggcgagggcgagggccgcccctacgagggcacccagaccgccaagctgaaggtgaccaagggtggccccctgcccttcgcctgggacatcctgtcccctcagttcatgtacggctccaaggcctacgtgaagcaccccgccgacatccccgactacttgaagctgtccttccccgagggcttcaagtgggagcgcgtgatgaacttcgaggacggcggcgtggtgaccgtgacccaggactcctccctgcaggacggcgagttcatctacaaggtgaagctgcgcggcaccaacttcccctccgacggccccgtaatgcagaagaagaccatgggctgggaggcctcctccgagcggatgtaccccgaggacggcgccctgaagggcgagatcaagcagaggctgaagctgaaggacggcggccactacgacgctgaggtcaagaccacctacaaggccaagaagcccgtgcagctgcccggcgcctacaacgtcaacatcaagttggacatcacctcccacaacgaggactacaccatcgtggaacagtacgaacgcgccgagggccgccactccaccggcggcatggacgagctgtacaagTAA
**ecRESCUE**	ATGgagaagcccctgctgcctaacgtgtataccctgaagcacaagttcttttggggcgccttcctgaatatcgcccggcacaacgcctttatcaccatctgccacatcaatgagcagctgggcctgaaaacccccagcaacgacgataagatcgtggacgtggtgtgcgagacatggaacaatatcctgaacaatgaccacgatctgctgaagaagtcccagct

	gaccgagctgatcctgaagcacttcccttttctgacagccatgtgctaccaccccccctaagaaggagggcaagaa<br>gaagggccaccagaaggagcagcagaaggagaaggagagcgaggcacagtcccaggcagaggccctgaac<br>ccatccaagctgatcgaggccctggagatcctggtgaatcagctgcactctctgGCAaactactatagcGCCt<br>ataagcacaagaagcccgacgccgagaaggatatcttcaagcacctgtataaggccttcgacgcctctctgagaa<br>tggtgaaggaggattataaggcccacttcaccgtgaatctgacaagggactttgcccacctgaaccgcaagggca<br>agaacaagcaggacaatcccgacttcaacagatacagattcgagaaggatggcttctttaccgagtctggcctgct<br>gttctttacaaatctgtttctggacaagagggatgcctattggatgctgaagaaggtgtccggcttcaaggcctctca<br>caagcagcgcgagaagatgaccacagaggtgttttgcaggagccgcatcctgctgcccaagctgaggctggagt<br>cccgctacgaccacaaccagatgctgctggatatgctgtctgagctgagcagatgtcctaagctgctgtatgagaa<br>gctgagcgaggagaataagaagcacttccaggtggaggccgacggctttctggatgagatcgaggaggagcag<br>aacccattcaaggacaccctgatccggcaccaggatagattcccctactttgccctgaggtatctggacctgaatga<br>gtccttcaagtctatccgctttcaggtggatctgggcacataccactattgtatctacgacaagaagatcggcgatga<br>gcaggagaagaggcacctgacccgcacactgctgtccttcggccggctgcaggactttaccgagatcaacagac<br>cccaggagtggaaggccctgaccaaggacctggattacaaggagacatccaatcagcctttcatctctaagacca<br>caccacactatcacatcaccgacaacaagatcggctttcggctgggcacaagcaaggagctgtacccctccctgg<br>agatcaaggatggcgccaatagaatcgccaagtacccttataactccggcttcgtggcccacgcctttatctctgtg<br>cacgagctgctgcctctgatgttctaccagcacctgaccggcaagagcgaggacctgctgaaggagacagtgcg<br>gcacatccagagaatctataaggacttcgaggaggagcggatcaataccatcgaggatctggagaaggcaaacc<br>agggcagactgccactgggagcctttcccaagcagatgctgggcctgctgcagaataagcagcctgatctgtccg<br>agaaggccaagatcaagatcgagaagctgatcgccgagacaaagctgctgtctcacaggctgaacacaaagct<br>gaagagctccccaaagctgggcaagcggagagagaagctgatcaagacaggcgtgctggccgactggctggt<br>gaaggatttcatgcgctttcagcccgtggcctacgacgcccagaatcagcctatcaagtctagcaaggccaactcc

	accgagttctggtttatcaggcgcgccctggccctgtacggaggagagaagaataggctggagggctatttcaag<br>cagaccaacctgatcggcaacacaaatccacaccccttcctgaacaagtttaattggaaggcctgcaggaatctgg<br>tggatttctaccagcagtatctggagcagcgcgagaagtttctggaggccatcaagaaccagccatgggagccct<br>accagtattgcctgctgctgaagatccctaaggagaacaggaagaatctggtgaagggatgggagcagggagg<br>aatctctctgccacggggcctgtttaccgaggccatcagagagacactgtctgaggacctgatgctgagcaagcc<br>aatccgcaaggagatcaagaagcacggccgggtgggcttcatcagcagagccatcaccctgtactttaaggaga<br>agtatcaggataagcaccagagcttctacaatctgtcctataagctggaggcaaaggcaccactgctgaagcggg<br>aggagcactacgagtattggcagcagaacaagcctcagtctccaaccgagagccagaggctggagctgcacac<br>aagcgaccgctggaaggattacctgctgtataagagatggcagcacctggagaagaagctgcggctgtaccgga<br>atcaggacgtgatgctgtggctgatgaccctggagctgacaaagaaccacttcaaggagctgaacctgaattatca<br>ccagctgaagctggagaatctggccgtgaacgtgcaggaggccgatgccaagctgaaccctctgaatcagaccc<br>tgcccatggtgctgcctgtgaaggtgtaccctgccacagcctttggcgaggtgcagtaccacaagaccccaatca<br>ggacagtgtatatccgcgaggagcacaccaaggccctgaagatgggcaacttcaaggccctggtgaaggaccg<br>gagactgaatggcctgttctcttttatcaaggaggagaacgatacacagaagcacccaatcagccagctgcggct<br>gaggcgcgagctggagatctaccagtctctgagagtggacgccttcaaggagacactgagcctggaggagaag<br>ctgctgaataagcacacatccctgtcctctctggagaacgagtttagggccctgctggaggagtggaagaaggag<br>tatgccgccagctccatggtgacagacgagcacatcgccttcatcgccagcgtgGCcaatgccttctgcGCca<br>accagtaccccttctacaaggaggccctgcacgcccctatcccactgttcaccgtggcccagcccaccacagag<br>gagaaggatggcctgggaatcgcagaggccctgctgaaggtgctgagggagtactgtgagatcgtgaagtccc<br>agatcggatcccttcaactgcctccacttgaaagactgacactgggatcccagctgcatttaccgcaggttttagctg<br>acgctgtctcacgcctggtcataggtaagtttggtgacctgaccgacaacttctcctcccctcacgctcgcagaata<br>ggtctggctggagtcgtcatgacaacaggcacagatgttaaagatgccaaggtgatatgtgtttctacaggagcaa

	aatgtattaatggtgaatacctaagtgatcgtggccttgcattaaatgactgccatgcagaaatagtatctcggagatc<br>cttgctcagatttctttatacacaacttgagctttacttaaataacgaggatgatcaaaaaagatccatctttcagaaatc<br>agagcgagggggggtttaggctgaaggagaatatacagtttcatctgtacatcagcacctctccctgtggagatgcc<br>agaatcttctcaccacatgaggcaatcctggaagaaccagcagatagacacccaaatcgtaaagcaagaggaca<br>gctacggaccaaaatagaggctggtcaggggacgattccagtgcgcaacaatgcgagcatccaaacgtgggac<br>ggggtgctgcaaggggagcggctgctcaccatgtcctgcagtgacaagattgcacgctggaacgtggtgggcat<br>ccagggatcactgctcagcattttcgtggagcccatttacttctcgagcatcatcctgggcagcctttaccacgggg<br>accacctttccagggccatgtaccagcggatctccaacatagaggacctgccacctctctacaccctcaacaagcc<br>tttgctcacaggcatcagcaatgcagaagcacggcagccagggaaggcccccatattcagtgtcaactggacgg<br>taggcgactccgctattgaggtcatcaacgccacgactgggaagggagagctgggccgcgcgtcccgcctgtgt<br>aagcacgcgttgtactgtcgctggatgcgtgtgcacggcaaggttccctcccacttactacgctccaagattaccaa<br>gcccaacgtgtaccatgagacaaagctggcggcaaaggagtaccaggccgccaaggcgcgtctgttcacagcc<br>ttcatcaaggcggggctgggggcctgggtggagaagcccaccgagcaggaccagttctcactcacgcttcaact<br>gcctccacttgaaagactgacactggtgagcaagggcgaggaggataacatggccatcatcaaggagttcatgc<br>gcttcaaggtgcacatggagggctccgtgaacggccacgagttcgagatcgagggcgagggcgagggccgcc<br>cctacgagggcacccagaccgccaagctgaaggtgaccaagggtggccccctgcccttcgcctgggacatcct<br>gtcccctcagttcatgtacggctccaaggcctacgtgaagcaccccgccgacatccccgactacttgaagctgtcc<br>ttccccgagggcttcaagtgggagcgcgtgatgaacttcgaggacggcggcgtggtgaccgtgacccaggactc<br>ctccctgcaggacggcgagttcatctacaaggtgaagctgcgcggcaccaacttcccctccgacggccccgtaat<br>gcagaagaagaccatgggctgggaggcctcctccgagcggatgtaccccgaggacggcgccctgaagggcg<br>agatcaagcagaggctgaagctgaaggacggcggccactacgacgctgaggtcaagaccacctacaaggcca<br>agaagcccgtgcagctgcccggcgcctacaacgtcaacatcaagttggacatcacctcccacaacgaggactac

	accatcgtggaacagtacgaacgcgccgagggccgccactccaccggcggcatggacgagctgtacaagatc<br>agtctgattgcggcgttagcggtagattacgttatcggcatggaaaacgccatgccgtggaacctgcctgccgatc<br>tcgcctggtttaaacgcaacaccttaaataaacccgtgattatgggccgccatacctgggaatcaatcggtcgtccg<br>ttgccaggacgcaaaaatattatcctcagcagtcaaccgagtacggacgatcgcgtaacgtgggtgaagtcggtg<br>gatgaagccatcgcggcgtgtggtgacgtaccagaaatcatggtgattggcggcggtcgcgttattgaacagttct<br>tgccaaaagcgcaaaaactgtatctgacgcatatcgacgcagaagtggaaggcgacacccatttcccggattacg<br>agccggatgactgggaatcggtattcagcgaattccacgatgctgatgcgcagaactctcacagctattgctttgag<br>attctggagcggTAA
